# Gum health and quality of life—subjective experiences from across the gum health-disease continuum in adults

**DOI:** 10.1186/s12903-022-02507-5

**Published:** 2022-11-18

**Authors:** Tom Broomhead, B Gibson, CR Parkinson, MV Vettore, SR Baker

**Affiliations:** 1 Unit of Oral Health, Dentistry and Society, School of Clinical Dentistry, University of Sheffield, Sheffield, UK; 2 Haleon, Weybridge, UK; 3 Department of Health and Nursing Sciences, Universitet i Agder, Kristiansand, Norway

**Keywords:** Gum health, Gingivitis, Periodontitis, Patient outcomes, Oral health, Quality of life

## Abstract

**Background:**

There has been a lack of qualitative work investigating the effects of the wide range of gum-related symptoms, and the perceived everyday impacts associated with these including on quality of life. While periodontal disease has been shown to have significant effects on quality of life, fewer studies have researched the perceived impacts of gingivitis and symptoms from across the entire gum health-disease continuum, despite evidence that these can also negatively affect quality of life. The aim of this study was to investigate perceived everyday impacts and explore the subjective experiences of adults with a variety of symptoms from across the self-reported gum health-disease continuum, and how these may affect quality of life.

**Methods:**

Participants were recruited at a large UK University using purposive sampling, for self-reported symptoms ranging from mild gingivitis to severe periodontal disease. Semi-structured interviews gathered details on symptom history, changes occurring over time and associated beliefs, as well as perceived impacts on everyday life, and links between these experiences and identity. Interviews were analysed using framework analysis based on the Wilson and Cleary health-related quality of life model.

**Results:**

Twenty-seven participants were recruited − 15 with symptoms of gingivitis, 12 with more severe periodontal symptoms. Prominent themes included description of symptoms, changes in daily life, social impacts, psychological impacts, identity, and overall impacts and quality of life. Differences were noted in severity, extent and frequency of symptoms and participant experiences, with greater perceived impacts often felt by those with periodontal disease. However, participants from across the gum health-disease continuum often expressed similar experiences and concerns.

**Conclusion:**

Findings demonstrate the range of experiences from participants with a variety of gum-related symptoms; notably, gingivitis was reported to have a range of perceived impacts on quality of life alongside those reported by periodontal disease sufferers. Future work should look to include symptoms from across the entire gum health-disease continuum when considering quality of life, as well as considering a more patient-centred approach which could be valuable in both clinical and research settings.

**Supplementary Information:**

The online version contains supplementary material available at 10.1186/s12903-022-02507-5.

## Background

Periodontal disease can significantly affect quality of life [1–4], with increased impairment with greater severity and extent [5]. Physical discomfort, psychological discomfort, psychological disability, social disability, and physical disability can be affected [6], alongside pain and functional limitations [7]. Gingival recession [8], gingival pockets and attachment loss [9] can also contribute to poorer oral health-related quality of life (OHRQoL), while appearance, self-esteem and overall health can be impacted by more severe symptoms [10]. Ageing can however be associated with perceptions of oral problems being less harmful, while the gradual state of attachment loss can allow time to adapt [7].

Fewer studies have explored patient’s perceptions of the effects of gingivitis. Yet, research suggests gingivitis can negatively affect quality of life across age ranges [11], particularly children and adolescents [12], impacting oral symptoms, functional limitations, and emotional and social wellbeing [13]. Gingivitis can also impact quality of life in adults [14, 15], particularly pain, difficulties brushing, and wearing dentures [7]. In addition, gingival treatment can be beneficial for quality of life [16], while increases in negative perceptions of quality of life among an elderly cohort with gingival problems have also been noted [17].

Previous research has focused less on everyday experiences of gingivitis, and symptoms from across the whole gum health-disease continuum, despite evidence that clinically less severe symptoms can affect everyday life [11]. Studies have often used broad clinical classifications [2] which may not accurately reflect the continuum of symptoms and experiences. While clinical assessments are vital, the importance of patient identified needs has also been emphasised, particularly for appropriate communication and treatment plans [7]. Furthermore, developing a patient-centred approach for understanding and, in turn, assessing everyday perceived impacts of gum health on OHRQoL is an important next step in clinical research and practice. In line with this, this study’s aim was to explore in-depth individual’s subjective experience of gum disease (in adults) including types and severity of perceived impacts along the gum health-disease continuum, and how these may be associated with quality of life, in order to inform the later development of a person-centred gum health quality of life measure. In this paper we adapt the ‘holistic’ term ‘gum health’ to be inclusive of all conditions that affect the gums. The ‘gum health-disease continuum’ therefore includes all symptoms associated with gum health, from mild symptoms such as light bleeding associated with gingivitis, through to heavier bleeding and soreness, and on to more severe symptoms such as gum recession, pain, and eventually loose teeth and tooth loss associated with periodontal disease (and all symptoms in between). The rationale for this was to help gain an understanding of the impacts of the full range of gum related symptoms, rather than focusing on a small sub-set.

## Methods

Participants were recruited from a large UK University through volunteer email lists using purposive sampling. The aim was to gather a range of experiences from different demographic backgrounds (age, gender, and socio-economic group according to NS-SEC category – National Statistics Socio-Economic Classification – [18]), and to recruit between 25 and 30 participants. The initial sample size of 25–30 participants was expected to enable the research team to examine whether data saturation had been reached [19]. The University’s research ethics committee approved the study (application 022394).

Participants were recruited in two phases. The first involved experiences and symptoms associated with gingivitis, using the following header in the recruitment emails: *‘Do you experience bleeding when brushing your teeth, or inflamed, tender or reddened gums?’*. To ensure individuals with more severe symptoms were not recruited, screening questions were sent to determine if they: (1) had been diagnosed with periodontal disease; (2) had been treated for periodontal disease; (3) currently had symptoms of periodontal disease; (4) were currently undergoing orthodontic treatment (which can affect gum health – [20]). Anyone answering ‘yes’ was ineligible. Examples of treatment types were included in some of the follow up questions to aid participants in their responses. For example ‘root scaling and polishing’ and ‘root planing’ were included in question 2, and ‘receding gums, tooth mobility, sores in mouth, bad breath, pus between gums and teeth’ were included in question 3.

The second phase was aimed at individuals with symptoms of periodontal disease using the following header: *‘Do you experience loose teeth, pain or discomfort in your gums, receding gums, bad breath, red or swollen gums, or tooth loss?’*. This allowed for the exploration of the illness careers [21] of those with more severe symptoms. Screening questions included whether respondents: (1) currently wore dentures (which can affect gum health); (2) had experienced tooth loss due to decay (which can affect gums, but not due to gum health-related problems); (3) had only experienced bleeding or inflammation when brushing (i.e. gingivitis). Anyone answering ‘yes’ was ineligible. This two-stage process allowed for the inclusion of participants with symptoms ranging from occasional light bleeding, to more frequent bleeding and inflammation, as well as discomfort associated with these symptoms, more advanced symptoms such as receding gums (to varying extents) and pain, to severe outcomes including bad breath, loose teeth, and loss of teeth and bone structure.

Participants were emailed an information sheet and consent form, with written consent obtained on the day of the interview. Participants were given a small honorarium (£25). Interviews were arranged at times and places that suited participants—usually either conference rooms at the University, or appropriate spaces at or near their work. Semi-structured interviews were used to explore the participants’ experiences. Interviews typically started by asking participants to describe their gum health, how long they had been experiencing symptoms, and any stimuli. Other topics included participants’ personal history, perceptions, experiences and knowledge of their condition, the impact of their condition on everyday life and changes or limitations that occurred over time, and the relationship between their experiences and identity. An additional file demonstrates the types of questions asked [see ‘interview_guide.docx’]. Phrases such as ‘gum health’ were not used to avoid influencing participants’ wording, with symptoms initially referred to as ‘condition’. Interviews were designed to be open and flexible in switching between topics. Questions were repeated at times to elicit more detail or probed to explore underlying narrative details. Interviews were conducted by one member of the research team (TB), and were recorded and transcribed verbatim with identifying information removed.

**Fig. 1:**
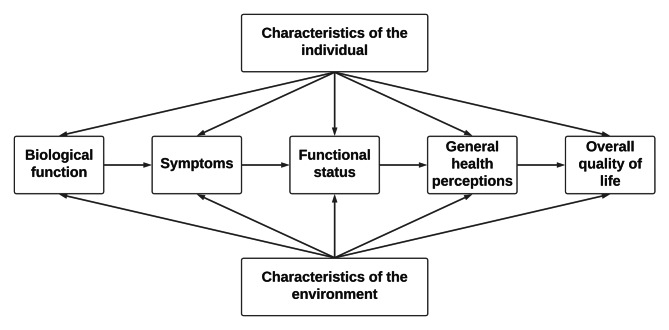
Wilson and Cleary Health-Related Quality of Life model (1995)

Transcripts were stored in NVIVO [22] and searched (and coded) for common themes using framework analysis [23] by TB, with an inductive, or bottom-up approach to framework analysis adopted. Following this, TB, BG, MV and SRB used this data to populate the Wilson and Cleary health-related quality of life model [24] (Fig. 1), which has been used previously to study impacts of oral health conditions [25]. This model links clinical variables with quality of life, and was selected based on its ability to accommodate functional, emotional and coping impacts associated with gum health symptoms. The framework’s focus is on understanding the relationship between clinical status, symptoms, functional limitations, and effects on general health perceptions and life overall. A further goal of using this model was to map the content of the interviews onto the appropriate domains in order to fully appreciate and analyse each item in turn, with themes and items that were identified and coded in Nvivo added continuously to the adapted and expanded model. From this, analysis of individual themes and items allowed for the emergence of broader, overarching domains which summarised the most important factors related to gum health. Themes and items were reviewed by the research team to ensure the quality of coding [26]. The results section is structured based on these overarching domains.

## Results

Twenty-seven participants were recruited − 15 from phase one (9 Female; 6 male) and 12 from the second phase (6 female, 6 male). Participant ages ranged from 23 to 73, with a mean age of 46. Socio-economic status, as measured by the NS-SEC showed a range, with 3.7% of participants being postgraduate students, or from ‘higher managerial and administrative’, ‘intermediate technical’, ‘semi-routine service’, ‘semi-routine clerical’ or ‘routine operative’ occupations, 7.4% from ‘lower managerial and administrative’ occupations, 11.1% from ‘intermediate clerical and administrative’ occupations, 18.5% from ‘higher professional traditional’ and ‘higher professional new’ occupations, and 22.2% from ‘traditional lower professional and higher technical’ occupations.

**Fig. 2:**
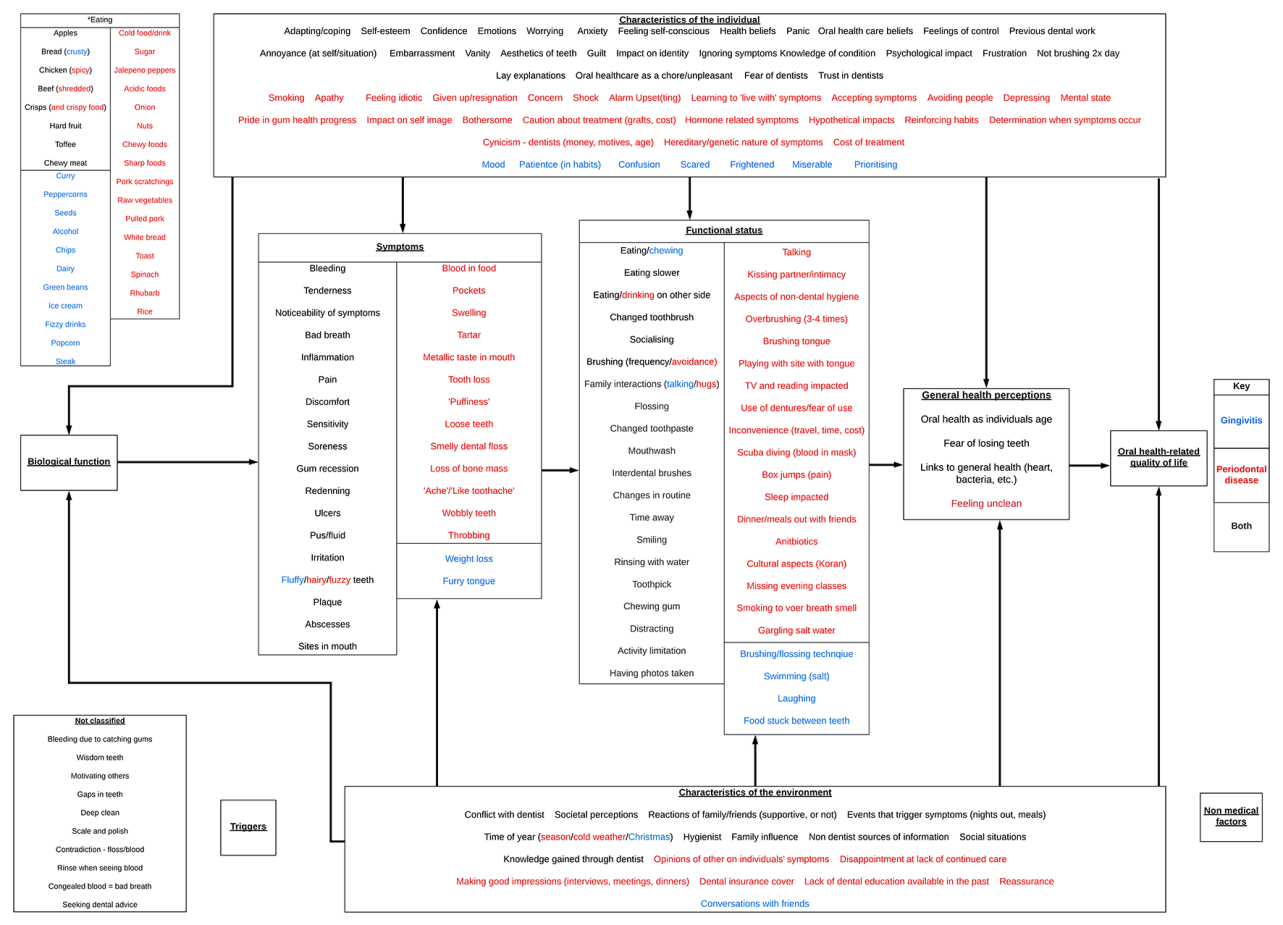
Modified Wilson and Cleary model populated with data from the interviews

The modified version of the Wilson and Cleary model [24] is presented in Fig. 2. Six main overarching domains emerged from the interviews: symptoms; changes in everyday life; social impacts; psychological impacts; identity; and overall impact and quality of life. The final domain (‘overall impact and quality of life’) is distinct from functional and social impacts, as it includes overall perceptions and concerns with gum health (overarching views and ratings of gum health for example) rather than functional and social impacts which are rooted in more specific contexts and examples (e.g. having to eat food more slowly, avoiding smiling or laughing around people). A summary of the most common and perceived impactful items from the framework can be seen below, with a selection of quotes to illustrate these experiences (Tables 1, 2, 3, 4, 5 and 6). Despite the wide variety of symptoms, for simplicity the results are presented separately for gingivitis participants (with less severe symptoms – bleeding, inflammation, tender gums) and periodontal participants (with more severe symptoms – pain, receding gums, bad breath, swollen gum, loose teeth).

### Symptoms

Bleeding was the most common self-reported symptom, with varying frequency, extent, duration, and locations where bleeding occurred. While concern varied, most participants recognised bleeding as a sign something was not right. Participants from both groups rationalised bleeding initially but became more concerned the longer it lasted. Common triggers included brushing and flossing. The visual nature of blood was concerning, even more so on rare instances when blood was seen on periodontal participants’ food in social situations. Several periodontal participants also described a bad or metallic taste when symptomatic. One periodontal participant described seeing pus in their gums, causing confusion and shock, while one gingivitis participant also reported the presence of pus. Gum recession was more often associated with increased symptom severity in periodontal participants, and sometimes attributed to oral health habits, ageing and wear and tear. Although several gingivitis patients reported gum recession this was less extensive. For some participants, particularly periodontal, recession proved a concern, given fears it could not be reversed.

Symptoms were occasionally referenced interchangeably (e.g. reddening, inflammation and swelling), or linked to other outcomes – for example, links between swelling and pus, pain, sensitivity, discomfort and reddening. Numerous sensations were described, including “sore”, “pain”, “irritated”, “sensitive”, “uncomfortable”, “tender”, and “throbbing”, sometimes together and sometimes interchangeably, with painful experiences likened to toothache on occasions by several periodontal participants. The presence of pockets, loose teeth, tooth loss and throbbing were all described by participants with more severe symptoms. Pain and discomfort were reported roughly equally by both groups, but sometimes used interchangeably with “discomfort” and “tender” among gingivitis participants. Unique symptoms for gingivitis participants included one-time mentions of ‘fluffy’ teeth, ‘furry’ tongue, and weight loss for one individual whose gingivitis stopped them eating for a short period. Additionally, fear of symptoms and progression was generally greater among gingivitis participants.

Although less common, bad breath was perceived to greatly impact periodontal participants who experienced it. For one participant this played on their mind in meetings, or when meeting new people at work, while another described its effect on everyday interactions such as hugging or having to face people when paying. Mechanisms to mask the smell included gum, mouthwash, extra deodorant and even smoking according to one periodontal participant, which was seen as preferable to anyone noticing bad breath. Some periodontal participants were able to accept severe symptoms such as loose teeth, citing hereditary factors and the conditions inevitability. While most participants with advanced symptoms were keen to avoid this stage, one periodontal participant described wanting to get loose teeth removed quickly to minimise impacts on their life.

**Table 1:** Example participant quotes—symptoms

Theme	Participant quote
Bleeding	‘I’ve got to admit at first I didn’t really think much about it, erm, but I think the longer it’s gone on the, the more I think I, I really could do with maybe doing something about it or, erm, but initially it was just a “oh dear”’ (Participant 1, Female, 53, gingivitis)
Blood on food	‘I can remember going for a curry once and eating poppadum’s and that happening, where there was blood on the poppadum and I was really embarrassed, but nobody noticed, so I got away with it’ (Participant 22, Female, 54, Periodontal disease)
Gum recession	‘So, it’s a bit worrying because it’s something that doesn’t come back, receding gums. It sounds like it doesn’t. You can’t pull them back up or anything. Once they’ve gone, they’ve gone that distance’ (Participant 27, Male, 45, Periodontal disease)
Bad breath	‘It’s like you, my little lad, you see him like turning away from me. You go for a cuddle and he like turns away from you because like your breath stinks. It honks, you can taste it in your mouth’ (Participant 17, Male, 47, Periodontal disease)
Loose teeth	‘Well, the tooth felt loose. I think both times I’ve had to go to the dentist to say, “can you take this out?” because I either was going on holiday or Christmas was coming up or something and I just thought, “This is going to cause me grief when I don’t need it” (laughs)’ (Participant 21, Male, 53, Periodontal disease)

### Changes in daily life

Participants from both groups avoided certain foods, or changed to foods requiring less chewing to avoid aggravating their gums, or getting food stuck between their teeth which led to further complications and symptoms. For gingivitis participants this included crusty bread, peppercorns, seeds, popcorn and steak, while periodontal participants mentioned nuts, pork scratchings, raw vegetables, toast, spinach and rice. Apples, chewy meats, crisps, hard fruits and toffee were mentioned by both groups. Other changes included where participants chewed in their mouth, often associated with flare-ups of symptoms, experienced by both groups but more commonly by periodontal participants. Several participants (two periodontal, one gingivitis) reported feeling that they ate more slowly when symptomatic to compensate, with one periodontal participant associating this with feeling self-conscious around others. Issues with chewing were only mentioned by gingivitis participants, while those with more severe symptoms were more likely to describe difficulties drinking in certain parts of their mouth.

Changing toothbrushes was slightly more common among periodontal participants, who mostly changed to electric brushes, which were generally seen as more effective at tackling symptoms, and occasionally softer brushes to ease stress on the gums. Similar patterns were seen with gingivitis participants, with one also switching to a firmer brush. Changes in technique were found in both groups. While some participants (from both groups) became more vigilant with brushing, others (more often gingivitis participants) felt they should avoid brushing parts of their mouth due to the associated pain, with one participant skipping brushing altogether at certain points. Mouthwash was used by participants in both groups, sometimes to ease or calm gum-related symptoms. Difficulties were noted in flossing by both groups, specifically keeping to routines, and pain and bleeding it brought on. Gingivitis participants seemed to experience less pain or soreness through flossing. Most knew they should floss regularly, with some participants from both groups citing its positive impact. Interdental brushes were seen to lead to less bleeding and were preferred by a number of participants, particularly those with more advanced symptoms.

**Table 2:** Example participant quotes—changes in everyday life

Theme	Participant quote
Changes in diet and eating	‘When I’ve forgotten to floss for a little while it does, it causes difficulty and I’ll opt for soups instead of, for example, chewier foods and toffees and things like that’ (Participant 10, Female, 23, Gingivitis) ‘It can be, erm, it can be a bit of a nightmare to eat on that particular side. That can happen with rough foods. That sort of thing…Yes, I would avoid eating anything on that side of the mouth’ (Participant 13, Male, 28, Gingivitis)
Change in brushing habits	‘The dentist said, “You brush this way, don’t you?” I said, “Yeah,” and she said, “No, you should brush this way because…”…Up and down, rather than lengthways, because lengthways what you’re doing is you’re brushing away some of the enamel on the top of your teeth where the tooth and the gum meets. You’re starting to get close to exposing more sensitive parts of the teeth – the internal part of the teeth’ (Participant 11, Male, 54, Gingivitis) ‘So that was worse. Er yeah, but like basically I just did what I normally do which is pay a bit more extra attention to it, brush a bit more in that area, and it did eventually clear up’ (Participant 3, Male, 27, Gingivitis)
Use of mouthwash	‘Yes, I would make sure that I cleaned more thoroughly and actually I said I didn’t use any other dental products, but when I got inflamed gums, I might use a mouthwash. I might use Corsodyl but only for a couple of days until the inflammation goes’ (Participant 4, Female, 64, Gingivitis)
Flossing and interdental brushes	‘Yeah, I thought it was fine and then they said do you floss and I said no, not really. If I’ve got something in my teeth then yes, but If I don’t, then no’ (Participant 2, Female, 30, Gingivitis) ‘Seeing the hygienist every 3 months, you know, when I got the usual telling off that I hadn’t been doing it right, all that kind of good stuff, and she pointed me out in the direction of some of the cleaning tools I wasn’t aware was available like the erm, the teepee brushes and stuff, er and gradually it’s kind of got under control’ (Participant 19, Male, 56, Periodontal disease)

### Social impacts

Despite being less common, issues related to social situations were still perceived to impact on participants. Some (more often periodontal participants) described being hesitant to smile at points to avoid others seeing their mouth or symptoms, with photographs being problematic. One gingivitis participant mentioned similar previous issues with laughing. Coping mechanisms were also cited, including one periodontal patient who covered their mouth with a hand in order to prevent anyone seeing their mouth, while another hesitantly talked to people at a 45-degree angle to conceal their breath. Concerns over talking and having to hide symptoms were present among gingivitis participants as well. Both gingivitis and periodontal participants had been concerned about other people noticing their symptoms and what others might think of these, with participants from both groups also having felt self-conscious about their condition at some point. One periodontal participant was concerned their symptoms could affect speech and pronunciation, while others felt conversation flow was interrupted due to pain, and some perceived that personal relationships and intimacy were affected by bad breath.

**Table 3:** Example participant quotes—social situations

Theme	Participant quote
Smiling	‘When I was younger, I’d probably feel a bit conscious about it…You know, smiling, laughing’ (Participant 9, Male, 44, Gingivitis)
Intimacy	‘It’s the bad breath. It’s so, I can’t tell you and your personal life with your wife, kissing—terrible. Because the effect on your partner is like, you don’t really want to kiss someone who’s got terrible, terrible breath. You don’t want to be up close with someone who has got terrible breath’ (Participant 17, Male, 47, Periodontal disease)
Talking	‘Only if it made my mouth look really a mess and, I was going somewhere…y’know where I’d got to actually y’know speak, or…erm… if…Normally if I were at my friends, they…who…we all know each other really well, it wouldn’t bother me… But maybe going to something where I was having to deliver a talk, or speak to somebody new or something like that. It might make me think…oh. I hate it when you’re talking to people and they’ve got their hand over their mouth and you can’t see what they’re saying’ (Participant 5, Female, 43, Gingivitis)
Noticeability of symptoms	‘I think probably my husband did notice years ago. And said, “Ooh, you need to sort that out.“ Or something, and then I think you feel a bit self-conscious or a bit silly that you haven’t done anything about it’ (Participant 12, Female, 39, Gingivitis)

### Psychological impacts

Many participants (from both groups) felt guilty or bad about their symptoms, sometimes due to infrequent flossing and interdental brushing, or not brushing properly or frequently enough. Some subsequently spent longer brushing than necessary at times to compensate (roughly equal numbers from each group). Some periodontal participants were concerned about the need for additional treatment, sometimes due to health implications, sometimes for financial reasons. Regarding the former, several participants worried about the irreversibility of symptoms, or the worsening of these, along with perceived effects on their quality of life.

Both groups expressed frustration and irritation at having to deal with symptoms and associated habits, with one gingivitis participant expressing frustration at letting their situation get to that point. Periodontal participants who had been less successful at treating symptoms believed it would be hard to improve the overall state of their gums. Despite most participants being able to carry on without symptoms affecting them, some participants perceived that it occasionally impacted their mood, sometimes for extended periods. One gingivitis participant described their ‘miserable’ experience, while several periodontal participants described their ‘depressing’ situation, with another noting how ‘fed up’ they got. Participants from both groups also reported feeling embarrassed when symptoms were visible or active. One gingivitis participant described feeling anxious about other people noticing, while others felt they had been self-conscious even in non-social situations. On occasions increased symptom severity was experienced alongside an air of resignation regarding symptoms and their consequences.

**Table 4:** Example participant quotes—psychological and emotional impacts

Theme	Participant quote
Oral hygiene habits	‘It can be a sign that you’re not brushing your teeth properly, or at least that’s what somebody told me, or what I read there. So, then you feel guilty and you start to spend longer on them’ (Participant 12, Female, 39, Gingivitis)
Concerns over more treatment	‘I was concerned. The pain wasn’t nice, and it’s not… It makes it difficult to do other things, but also I was anxious about was I going to have to have some dental treatment that I wouldn’t like?’ (Participant 23, Female, 73, Periodontal disease) ‘And the dentist, yeah, I think she would probably suggest measures to deal with tooth loss that would be costly – costly and invasive’ (Participant 24, Female, 63, Periodontal disease)
Concern for future health	‘So, I don’t want that to happen too early. I really want to live a better quality of life in terms of my dental health. So, I thought I need to get it controlled. I know I cannot reverse what has happened’ (Participant 26, Female, 44, Periodontal disease) ‘I just worry that things may get worse in 10 years’ time or something…so I’m 50 now, what’s going to happen when I’m in my 60s? Am I going to have more problems? More visits to the dentist and this kind of thing’ (Participant 20, Female, 53, Periodontal disease)
Frustration and irritation	‘Not annoyed with myself, particularly given that it ended up not being a traumatic experience; just a bit frustrated that I had let it get to this point’ (Participant 14, Female, 27, Gingivitis) ‘And it’s irritating, and when I do clean it with the brush the dentist has told me to I always make it bleed, and then it flares up’ (Participant 18, Female, 44, Periodontal disease)
Improving state of gums	‘Yeah, they can’t cure it, and it’s not going to stop it, just slow it down. And I think there is all just account to keep my teeth. That’s what I’d led to believe anyway by the dentist. We’ve done these various deep cleans and I’ve kept on brushing. I think that’s about as much, well, that’s what they tell me, as much as they can do’ (Participant 21, Male, 53, Periodontal disease)
Affecting mood	‘What did I do? Yeah, I sort of remember. I remember ploughing on at work for a while but being miserable with it. It was a good month. It was over a month’ (Participant 8, Male, 44, Gingivitis)
Embarrassment	‘Knowing that it means that there’s something wrong, it’s a bit of an embarrassing symptom because it’s not supposed to be there. I think with something like this, where it’s very much considered, “This wouldn’t happen to you if you had perfect oral hygiene,” you’re a bit like, “Well, I don’t want someone to know, then, do I, if this is happening, because it’s obviously something I’m doing wrong?”’ (Participant 14, Female, 27, Gingivitis)

### Identity

One periodontal participant felt their ‘horrible’ experience negatively affected their ‘self-image’. Comments from some periodontal participants on the perception that symptoms made them feel old aligned with previous statements from both groups on the advancement, or inevitability of symptoms with age, with some gingivitis participants noting associations with being neglectful, amid fears of losing teeth. Perceived activity limitations were noted, more commonly among periodontal participants, including physical activities and daily routines such as work and reading. While most felt their identity and symptoms were separate, some accepted their condition was now part of their identity, with one gingivitis participant linking this to their smile. Acceptance varied from several periodontal participants who seemed slightly disappointed about this, to those who seemed to embrace it, as it represented a healthy mouth or overcoming symptoms or fears. Similarly, while some seemed sad and resigned about their condition, other (often-older) participants were able to accept their situation and tried to get on with their lives.

**Table 5:** Example participant quotes—identity

Theme	Participant quote
Self-image	‘It’s horrible I think what happened to me is horrible, it affects my image of myself, I think my self-image’ (Participant 26, Female, 44, Periodontal disease)
Feeling old	‘I thought “oh gosh I’m going to lose all my teeth”. I’m going to be like, my grandad with false teeth or something. It horrifies me, it’s the association with being old and neglectful and things like that’ (Participant 5, Female, 43, Gingivitis)
Acceptance	‘It’s one of those things, you go “Oh”, and that’s it. You know you can’t do anything about it, as I was saying, when I lost that one. I feel too old to bother now, that’s one of the things. If I was a lot younger, yeah’ (Participant 25, Male, 65, Periodontal disease)
Part of identity	‘Yeah. It is a difficult one, isn’t it? I have to think of it as being who I am because it’s something I’ve got to accept’ (Participant 20, Female, 53, Periodontal disease) ‘Well, I guess so. I mean, you know, I guess your teeth are how you smile a lot so, you know, I’ve got quite decent teeth and it’s had benefits, I guess, because, you know, I know it’s linked to my parents and stuff like that. So, I’m quite pleased that they’re okay. That’s why I’d be worried if they got discoloured or if I had to have teeth out or that kind of stuff. So, I can see the relationship there’ (Participant 15, Male, 35, Gingivitis)

### Overall impacts and quality of life

Fearing tooth loss at some point was common across the continuum, sometimes for aesthetic reasons, while some periodontal participants worried about functional limitations, difficulty eating and quality of life. Most understood this was a potential consequence of advancing symptoms, although those with less severe symptoms were often more afraid of this. Concerns that gum problems could be serious affected participants from both groups. Some periodontal participants described feeling unclean or unhealthy when discussing symptoms, while participants from both groups referenced general perceptions of unhealthiness and having symptoms. Links were also made to other health conditions such as heart disease by several periodontal participants. Some participants (from both groups) saw their symptoms as part of their overall health, with one gingivitis participant making associations with these and other conditions they experienced, describing the body as one system. Symptom severity and impact varied among participants, as did length of time and frequency at which these were experienced. Some had been dealing with symptoms for decades, while for some these had occurred only in recent months.

**Table 6:** Example participant quotes—overall impacts and quality of life

Theme	Participant quote
Fear of losing teeth	‘Well you think [in response to symptoms], “am I going to lose all my teeth?” and things like that. Vanity basically, I don’t want to lose all my teeth just yet’ (Participant 16, Male, 59, Periodontal disease) ‘I was upset really. I was panicking, I was thinking “oh dear am I going to lose that tooth”’ (Participant 6, Female, 51, Gingivitis)
Feeling unhealthy or unclean	‘But at the time it feels really rubbish. It makes me feel bad and sometimes it makes you feel like, it’s unclean, it’s that unclean thing’ (Participant 22, Female, 54, Periodontal disease) ‘I do think [of myself] as someone who hasn’t looked after themselves. Generally quite unhealthy. So, it probably complements that’ (Participant 27, Male, 45, Periodontal disease)
Links to wider health issues	‘I sometimes talk with my parents, er or family and friends about it, er, it could also happen, so for example if er, your general health is not in good condition this can cause kind of er, side effect er [ok] but erm [right] I’m not sure whether I am a case’ (Participant 7, Female, 40, Gingivitis)
The body as one system	‘She [Giulia Enders] wrote a book a few years back about the gut, and the microbiome, and things like this and everything. She says “it’s one system”, and that makes a lot of sense really. Why…? I think we need to be moving more towards holistic medicine. Treating one thing as one thing and another thing as another, you only go so far with solving problems if you do that, and I think having dentists separate does that’ (Participant 8, Male,44, Gingivitis)

## Discussion

This study aimed to investigate perceived everyday impacts and subjective experiences from across the gum health-disease continuum, and how these may affect quality of life. The findings demonstrate for the first time the sheer range of perceived impacts associated with symptoms, and functional, social, psychological and emotional aspects of everyday life. Use of the Wilson and Cleary conceptual model allowed for the systematic mapping of these signs and symptoms within one comprehensive framework. While the range of perceived impacts was generally greater among those with self-reported periodontal disease, participants with gingivitis were also shown to experience impacts in daily life. Consequently, a range of coping mechanisms and adaptations were seen. Some became familiar and comfortable in dealing with symptoms, while for others the perceived fear of advancement was clear, and was experienced alongside a sense of resignation among some for whom this had already occurred.

Similar to previous studies, participants with symptoms of periodontal disease expressed discomfort, perceived emotional impacts in having to deal with symptoms [6], and felt they had numerous functional limitations [7]. Appearance and self-esteem were also important considerations for some participants [10], with the stigma of periodontal disease [6] and associated symptoms leading to embarrassment for some. Expressions of guilt for failing to prevent symptoms was also in line with previous findings [27]. While some participants believed it would be hard to improve their gums, there was also a sense of acceptance and adaptation by older participants, who adjusted to their condition [7], as well as those who had to confront and manage symptoms. Links between gum symptoms and identity are less prevalent in the literature, and this study demonstrated that while most rejected such links, several participants now considered their condition to be part of who they were. This acceptance came with resignation from some and a sense of pride for others due to persevering with their treatment and changes they were able to achieve.

Although perceived gingivitis related impacts were sometimes less than in participants with periodontal symptoms, these still induced discomfort and pain, were felt to impact on functional aspects such as brushing and eating, and had perceived emotional (concern, worrying, feeling self-conscious) and social impacts (societal perceptions, embarrassment). These are in line with previous results [7, 13], however the finding that participants with less severe symptoms were often more fearful of their condition and advancement of symptoms than those with more severe symptoms has been explored less within the literature. This research also found a far wider range of perceived impacts than previous studies, demonstrating that all gum health conditions should be considered in OHRQoL related discussions.

Strengths of the research include use of semi-structured interviews to explore experiences of participants in a flexible and open-ended manner. Additionally, use of a person-centred approach was key in furthering understanding of the experiences that mattered most to participants, and can help inform new strategies (behavioural, communication and clinical). Previous studies have demonstrated that self-impression and self-awareness of oral health are highly associated with both periodontal presence and stages [28], and self-reported outcomes are considered central in understanding a given condition, and what it means to live with it [29]. Recruitment was also designed to capture participants with a wide range of gum-related symptoms. The study also has importance for clinicians in understanding patient perceptions and the need to improve their quality of life through oral care. The results of this study are also important in supporting the development of a disease specific OHRQoL measure that could represent the themes identified by this study. There were also some limitations. While data saturation was achieved, participants were recruited from a university setting, with demographic backgrounds not being representative of national data, and the applicability of these findings in other settings should be considered with caution. In addition, no inter-coder or intra-coder kappa agreement analysis was undertaken due to there only being one coder. More generally, the power dynamics of qualitative research and potential social desirability bias should be borne in mind [30], while emergence and consolidation of information from this type of research is inextricably bound with the knowledge, experiences and world views of the research team [27].

Further research incorporating more detailed accounts of gum-related symptoms in daily contexts (diary analysis) may clarify how processes related to gum symptoms are shaped and interact with everyday life. Creation of a gum-specific quality of life measure may also be beneficial in helping achieve a patient-centred approach and incorporating the types of perceived daily impacts and concerns found here. These findings lay the groundwork for such research. Many of the items in commonly used OHRQoL measures such as OHIP [31], OIDP [32] and GOHAI [33] are either not related to gum health, or are combined with impacts on the mouth and teeth. Additionally, while many items could relate to gum health, they do not do so explicitly, and it is likely they were not included based exclusively out of concern for gum health. Many of the domains from the aforementioned measures cover similar domains to those emerging from this qualitative research. For example, all seven of the OHIP domains (functional limitation, physical pain, psychological discomfort, physical disability, psychological disability, social disability, and handicap) have similar examples in this study’s data, as do the main themes of OIDP (eating, speaking, cleaning teeth, relaxing, showing teeth, emotional status, carrying out work, enjoying social contact) and GOHAI (limiting food, trouble biting/chewing, speaking, discomfort, limiting contact, appearance, worry/concern, feeling nervous or self-conscious, uncomfortable eating in front of others, sensitivity to hot/cold foods). However, these measures do not include questions on how oral health conditions may affect personal concerns such as identity, feelings of guilt, financial concerns, worries over symptom progression, or the range of adaptations in oral hygiene routines that are made to accommodate symptoms. They also do not account for the range of detailed gum-specific symptoms, everyday impacts and individual feelings towards symptoms that were identified in this research. Overall, this demonstrates that current measures do not consider all aspects relevant to, and related to gum health, and that using the findings of this research to develop a gum-specific measure may be of value to both patients and clinicians.

## Conclusion

This research demonstrates the wide range of perceived impacts and experiences from across the gum health-disease continuum, which were felt to affect a range of social, functional and psychological aspects of everyday life. While greater impacts were usually experienced by those with more severe symptoms, gingivitis was perceived to impact the quality of life of some participants, who also experienced unique impacts from their symptoms. These findings demonstrate the benefits of person-centred approaches to gum health-related research, which could be valuable in clinical settings in aiding with communication and finding appropriate treatment plans.

## Electronic supplementary material

Below is the link to the electronic supplementary material.

Additional File 1: Interview Guide.

## Data Availability

The datasets used and/or analysed during the current study are available from the corresponding author on reasonable request, on condition that confidentiality of the data is maintained.

## List of abbreviations

OHRQoL: Oral health-related quality of life
NS-SEC: National Statistics Socio-Economic Classification
OHIP: Oral Health Impact Profile
OIDP: Oral Impacts on Daily Performance
GOHAI: Geriatric Oral Health Assessment Index
