# Supplementary material for: Gum health and quality of life—subjective experiences from across the gum health-disease continuum in adults
Source: BMC Oral Health. 2022 Nov 18;22:512. doi: 10.1186/s12903-022-02507-5 (PMC9675234; doi:10.1186/s12903-022-02507-5)
Supplement: Supplementary file 1 — Additional File 1: Interview Guide. [file 12903_2022_2507_MOESM1_ESM.docx]

Appendix A – Interview guide

**Interview guide**

**Interview guide for impact study on gum-related quality of life.**

This guide is designed to help probe and explore different aspects of gum-related health. Start by screening (although you should also try finishing with those questions because they might impact on the flow of the interview). The key is to start broad by asking participants to tell you about their experiences of gum health, then focus on the details of each of the issues as they arise.

***Note we are also seeking to work from gum health in the broadest sense so in this guide we are avoiding using gum health as a term for the interviews. There will be those who will use the term and those who don't. It is important to monitor carefully the language being used in the interviews and to listen carefully to the participants and see if they use the term.***

***Initially, at the recruitment stage we used the terms ‘loose teeth, pain or discomfort in your gums, receding gums, bad breath, red or swollen gums, or tooth loss’.***

***Further, the respondent's description used to discuss their gum health which is otherwise referred to as ‘condition’ in this guide (to be used by the researchers)***

If some issues do not arise naturally then probe for them in turn by starting general – much the same as previous interviews you have conducted.

Acknowledge (if appropriate) that you appreciate that this can be a very private experience, and that you’re grateful they’re sharing their experiences with you.

**Open the interview by screening the range of impacts the participant is experiencing.**

Ask if any of the following stimuli aggravate their condition.

- tooth brushing

- flossing

- eating certain foods (i.e. apples, hard foods)

Ask if they:

- have been to their dentist about this

- use a special toothbrush or toothpaste as a result of their experience.

**1. Explore their personal history, perceptions, experience and knowledge of the condition**

This is a guide for the topics to be covered in the interview, try by opening with a very general question such as ‘you say you’ve had problems with your gums. Could you tell me a bit more about it?’. Specifically, you are looking for:

Experience of the onset and biographical aspects of the condition

When they first noticed the symptoms of interest?

Or how they became aware of it?

Explore their initial reactions to the condition (effect, location, response)

Specifically ask about the first incident.

How did it affect them the first time it happened? Were they concerned?

Did they experience any pain?

What were they doing at the time?

What did they do afterwards, or as a result?

Did anyone else notice? How did that make them feel?

When asking about the illness career explore (length and changes in the condition)

How frequently are they experiencing these incidents?

Among the symptoms that triggers the condition, which do they think causes it most?

Since the symptoms have started, have they tended to be the same each time they have occurred?

Can they tell you about these changes if there were any?

Could they tell you about the worst incident they experienced?

Why was it worse on this occasion?

Do these incidents tend to occur in a specific area of the mouth? Or in different areas?

If in different sites, ask them if they can compare experiences across these different sites?

What have they done about these incidents?

[For ignorers] What would make them do something about it?

Explore their understanding about their condition. We are looking for lay beliefs (knowledge) and their emotional reactions to their condition, *please bear in mind if they do not use the term gingivitis/gum health to describe it they may not know much about it*.

What do they know about the condition in general? How did they find out about this?

How would they usually look after their oral health? (no prompt, so as not to influence answer)

Would they usually use any other oral hygiene products?

Have they thought about why they might be experiencing these symptoms? Why do they think this has happened?

What is their emotional reaction to these incidents?

How difficult is the condition to experience?

**2. Explore the impact of the condition on their everyday life. The categories we are looking at are limits in activities and participation. We also need to know about the emotional burden if any. We are also seeking information about any adaptation and coping strategies and their perceptions of treatment.**

Specifically, you need to look at:

Activity limitations (eating, chewing, tooth brushing, other functional limitations) – probing on commonly associated phenomena:

How do the symptoms bother them in everyday life?

Do they prevent:

Eating?

Brushing?

Flossing?

Other activities?

Participation restrictions (social restrictions, situational burden)

What are the situations in which they are likely to start thinking about the symptoms?

Can they ‘forget’ about it when socialising?

Do the symptoms affect everyday interactions? (e.g. smiling, talking)

Can they give examples of situations where it has become a particular problem?

Have they told other people that they have got this condition? If so why?

- If they have not told anyone please find out why.
- What do others think of what they experience?

Emotional burden

If there is anything they cannot do because of the symptoms, how does that make them feel about it?

Does it worry them?

Has the extent to which it worries them changed?

Adaptation and coping strategies

Is it possible to predict what might make the symptoms worse?

Do they anticipate, the symptoms getting worse when eating, etc?

Do they have to adapt the way they eat certain foods?

Have they had to adapt the way they brush?

What do they do to prevent the symptoms?

What do they do when it comes?

Prevention and treatment (self-care, dental care)

Have they used any different products to help with their gums?

Which products are these?

Have they made a difference? If so how does this help?

What did you discuss about your condition with the dentist?

**3. The relationship between their identity and experience of having this condition**

Focus on the condition in the context of their lives, has it changed anything, made differences in the way they live

If they think of the symptoms do they change the way they think about their teeth, their health in general, about themselves?

Are they a problem or a nuisance in their life?

- If they have said it is a part of who they are now – try to explore this in more depth, in other words ask what that means.
